# Annotation and characterization of the *Plasmodium vivax* rhoptry neck protein 4 (*Pv*RON4)

**DOI:** 10.1186/1475-2875-12-356

**Published:** 2013-10-05

**Authors:** Gabriela Arévalo-Pinzón, Hernando Curtidor, Jesica Abril, Manuel A Patarroyo

**Affiliations:** 1 Fundación Instituto de Inmunología de Colombia FIDIC, Carrera 50 # 26-20, Bogotá, Colombia; 2 Universidad del Rosario, Carrera 24 # 63C-69, Bogotá, Colombia

**Keywords:** Bioinformatics analysis, Invasion, *Plasmodium vivax*, Rhoptry neck protein, Rhoptry, Tight junction

## Abstract

**Background:**

The tight junction (TJ) is one of the most important structures established during merozoite invasion of host cells and a large amount of proteins stored in *Toxoplasma* and *Plasmodium* parasites’ apical organelles are involved in forming the TJ. *Plasmodium falciparum* and *Toxoplasma gondii* apical membrane antigen 1 (AMA-1) and rhoptry neck proteins (RONs) are the two main TJ components*.* It has been shown that RON4 plays an essential role during merozoite and sporozoite invasion to target cells. This study has focused on characterizing a novel *Plasmodium vivax* rhoptry protein, RON4, which is homologous to *Pf*RON4 and *Pk*RON4.

**Methods:**

The *ron4* gene was re-annotated in the *P. vivax* genome using various bioinformatics tools and taking *Pf*RON4 and *Pk*RON4 amino acid sequences as templates. Gene synteny, as well as identity and similarity values between open reading frames (ORFs) belonging to the three species were assessed. The gene transcription of *pvron4*, and the expression and localization of the encoded protein were also determined in the VCG-1 strain by molecular and immunological studies. Nucleotide and amino acid sequences obtained for *pvron4* in VCG-1 were compared to those from strains coming from different geographical areas.

**Results:**

*Pv*RON4 is a 733 amino acid long protein, which is encoded by three exons, having similar transcription and translation patterns to those reported for its homologue, *Pf*RON4. Sequencing *Pv*RON4 from the VCG-1 strain and comparing it to *P. vivax* strains from different geographical locations has shown two conserved regions separated by a low complexity variable region, possibly acting as a “smokescreen”. *Pv*RON4 contains a predicted signal sequence, a coiled-coil α-helical motif, two tandem repeats and six conserved cysteines towards the carboxy-terminus and is a soluble protein lacking predicted transmembranal domains or a GPI anchor. Indirect immunofluorescence assays have shown that *Pv*RON4 is expressed at the apical end of schizonts and co-localizes at the rhoptry neck with *Pv*RON2.

**Conclusions:**

Genomic, transcriptional and expression data reported for *Pv*RON4, as well as its primary structure characteristics suggest that this protein participates in reticulocyte invasion, as has been shown for its homologue *Pf*RON4.

## Background

Malaria is a parasitic disease, primarily affecting pregnant females and children aged less than five years in tropical and subtropical areas around the world, mainly in sub-Saharan Africa [1]. A substantial increase in cases of *Plasmodium vivax* malaria has been reported recently in Asia and Latin America [2]*.* Such data, added to an increase in the number of severe disease reports, death and resistance to first-generation anti-malarial drugs attributed to *P. vivax*[3-5], emphasize the need for developing effective control tools and acknowledging this parasite species as an important agent of vector-transmitted tropical diseases.

Basic research around the world into *P. vivax* has lagged behind compared to *Plasmodium falciparum* mainly due to a lack of *in vitro* continuous culturing as this requires a large amount of reticulocytes for maintaining and propagating this parasite species [6]. Among the various strategies being used to overcome this problem, comparative analysis with other species, such as *P. falciparum* and adapting *P. vivax* strains to *Aotus* monkeys [7], have allowed the identification during the last few years of several antigens in *P. vivax* which could be participating in invasion [8-11].

The merozoite’s rapid and coordinated invasion of red blood cells (RBC) is mediated by initial contact with the host cell, re-orientation of the parasite’s apical pole and active internalization into the host cell involving many sub-compartmentalized proteins localized in secretory organelles, such as micronemes and rhoptries [12]. The rhoptries are pear-shaped structures having two compartments called neck and bulb; despite being present in all parasites belonging to the phylum *Apicomplexa*, their size and number varies according to the target cell being invaded [13]. Transmission electron microscopy studies of *P. falciparum* and *Toxoplasma gondii* have revealed that some antigens are site-specifically distributed in a rhoptry [14,15]; such distribution seems to be the rule concerning important organization facilitating protein function in tight junction (TJ) and/or parasitophorous vacuole (PV) formation. Proteomics analysis of *T. gondii* rhoptries has shown minimum overlapping with *P. falciparum* proteins, except for some proteins localized in the rhoptry neck (RON), which could be involved in functions common to both parasites [16]. RON2 [17], RON4 [18] and RON5 [19] proteins have been identified to date in *P. falciparum*, forming part of a high molecular weight complex interacting with a microneme-derived protein called apical membrane antigen 1 (AMA-1) [20]. The assembly of this macromolecular complex allows TJ formation or irreversible binding which is important since this acts as a foothold for the parasite’s motor machinery to propel the parasite into the PV for successful invasion.

Surface plasmon resonance (SPR) studies have determined that the *Pf*RON2 binding region to *Pf*AMA-1 lies within residues 2,020-2,059 and contains two conserved cysteines which are strongly implicated in the peptide’s structure [21]. Crystallization of the *Pf*AMA-1/*Pf*RON2 complex has shown that the peptide binds in a U-shape to the AMA-1 ectodomain, inducing a conformational change in AMA-1 domain II (DII) [21], similar to that found for the interaction between *Tg*AMA-1/*Tg*RON2 [21]. Interestingly, different methodological approaches have proposed that blocking the interaction between AMA-1 and RON2 inhibits parasite invasion of its host cell [20,22,23]. Knowledge regarding these interactions has been key in understanding 4G2 antibody’s invasion-inhibitory action (binding to AMA-1 DII), thereby impeding displacement in domain II and thus blocking interaction with RON2 [21].

*Pf*RON4 is the second most studied antigen from the aforementioned complex after AMA-1 and RON2. This is one of the most important proteins as it is most likely essential in merozoite and sporozoite invasion of their respective host cells [24]. No evidence has been advanced to date regarding the role played by RON4 in TJ formation and it is not known which proteins interact directly with it within the complex. However, RON4 has been the only protein from the whole complex that has been convincingly localized to junction constriction during invasion [25]. In fact, immunoprecipitation assays with *Plasmodium yoelii* merozoite proteins have supported RON4 (*Py*P140)-AMA1 interaction but *Py*RON2 and *Py*RON5 have not been identified within the complex. Importantly, immunogenicity studies have shown that *Pf*RON4 sequences elicit immunogenic responses in natural human malaria infection, suggesting their exposure to the immune system during host cell invasion as has been proposed for other important antigens, such as merozoite surface proteins (MSP) [26].

The importance of RON proteins in *P. falciparum* and in the model *Apicomplexa* parasite, *T. gondii*, has led to interest in identifying the functional characterization of RON proteins in other genera, such as *Neospora*, *Cryptosporidium*, *Babesia,* and other *Plasmodium* species [8,27]. Recent studies have shown that although most proteins from the complex are conserved within *Apicomplexa*, some are exclusive to certain members [27]. Such divergence regarding the complex’s molecular composition suggests differences in TJ formation among *Apicomplexa* members and partly explains host cell specificity among parasites [27].

This study was thus aimed at making a correct annotation for *pvron4* in the *P. vivax* genome and characterizing *Pv*RON4 in late-stage schizonts from the VCG-1 strain. *Pv*RON4 is a 733 amino acid long protein containing typical characteristics concerning antigens involved in invasion, such as having a signal peptide, coiled-coil α-helical motifs, low complexity regions, and long conserved segments. Immunofluorescence studies using rhoptry and membrane markers as reference have shown that *Pv*RON4, just like its homologue in *P. falciparum* (*Pf*RON4), is localized in the rhoptries and could thus be participating in invasion.

## Methods

### Bioinformatics tools

The Basic Local Alignment Search Tool (BLAST), from the National Center for Biotechnology Information (NCBI), was used to find the homologous gene to *pfron4* (PF11_0168) and *pkron4* (PKH_091340) in the *P. vivax* genome. The presence of and boundaries between *pvron4* introns and exons was evaluated using Genscan [28], Spidey [29] and tBlastn. Gene structure, open reading frame (ORF) transcription direction, nucleotide and amino acid level identity, and similarity values between *P. falciparum*-*P. vivax* and *Plasmodium knowlesi*-*P. vivax* were taken into account for synteny analysis.

Bioinformatics tools, such as SignalP [30], were used for evaluating the presence of a signal peptide in *Pv*RON4 sequence; TMHMM, Polyphobius and PredGPI were used to predict transmembranal domains and glycosylphosphatidylinositol (GPI) anchors, since the presence of these motifs and domains is typical of several antigens considered as candidates for an anti-malarial vaccine [31-33]. Sequence tandem repeats extraction and architecture modelling (XSTREAM, variable 'X’) was used for finding repeat sequences and the simple modular architecture research tool (SMART) [34] with Globplot for searching for other important motifs and domains.

### DNA, RNA and protein source and cDNA synthesis

A blood sample taken from an *Aotus sp.* monkey infected with the Colombian *P. vivax* Guaviare 1 strain (VCG-1) was used for extracting nucleic acids and proteins. The VCG-1 strain had been cultured via successive passes *in vivo* in *Aotus sp.* monkeys from the primate station in Leticia, Amazonas, as thoroughly described [7]. The monkeys were kept under constant care, supervised by a primatologist, according to the conditions established by Colombian Animal Protection law (law 84/1989) and Corpoamazonía (the Colombian entity regulating environmental matters in the region) (resolution 00066, September 13, 2006).

A schizont-enriched sample was obtained using a discontinuous Percoll gradient (GE Healthcare) and genomic DNA (gDNA) was isolated using a Wizard genomic DNA purification kit (Promega), following the manufacturer’s recommendations. RNA was isolated from the parasite using the Trizol method [35], followed by treatment with RQ1 RNase-free DNase (Promega). Purified RNA and gDNA integrity was examined by electrophoresis on agarose gels. Five microlitres of RNA were taken as template for cDNA synthesis, using a one-step RT-PCR SuperScript III kit (Invitrogen).

### Primer design and *pvron4* amplification

**Figure 1:**
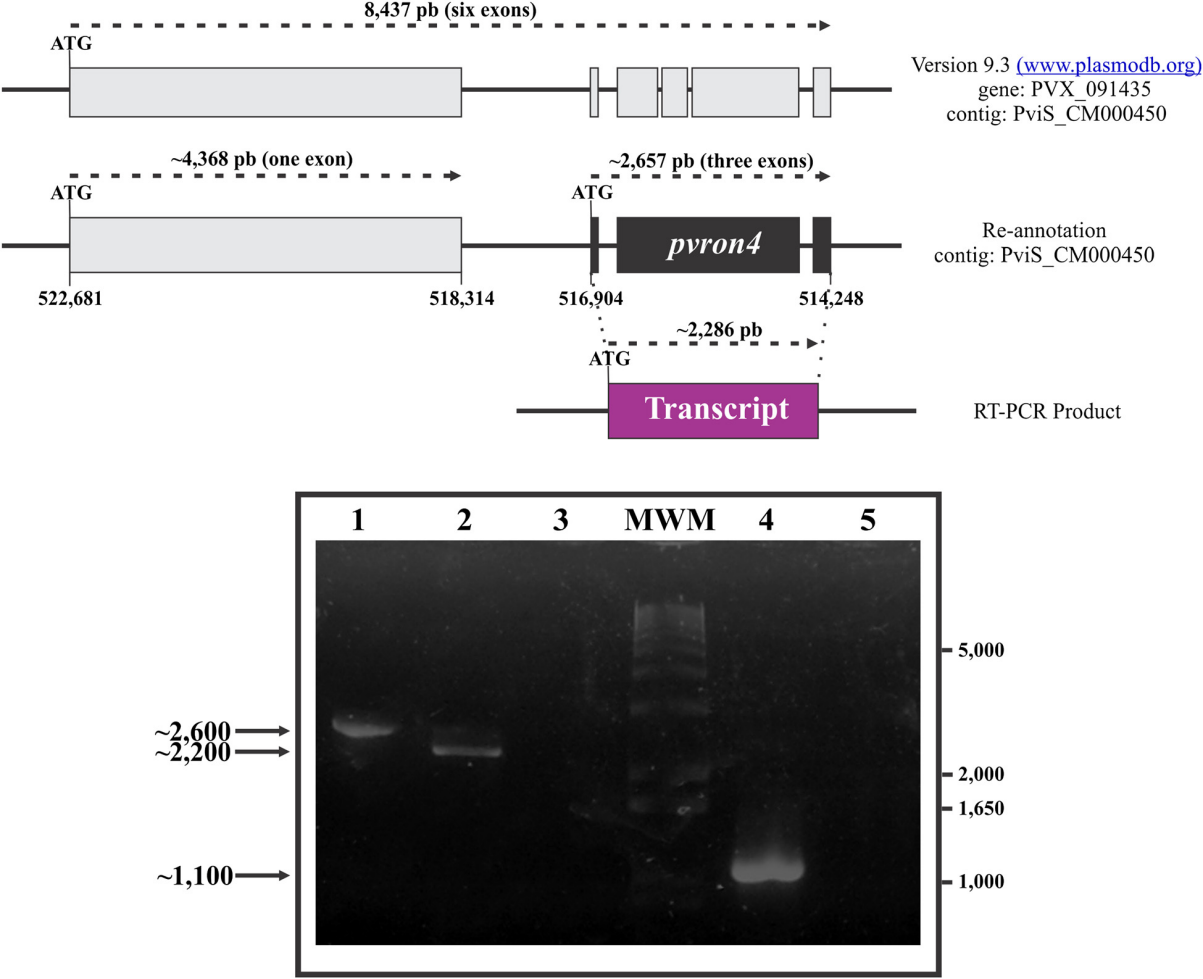
**Re-annotation of *pvron4 *within the PviS_CM000450 contig (above) schematic representation of PVX_091435 from the *pvron4 *gene in PlasmoDB and annotation suggested after bioinformatics and experimental analysis.** The start positions for the ORFs within the contig are shown and the transcription direction. PVX_091435 was originally annotated as an 8,437 bp gene consisting of six exons. The re-annotation proposed has two ORFs where *pvron4* start codon (shown in bold) is located 5,780 bp downstream the start codon initially reported in PlasmoDB. Amplification of the expected PCR products for *pvron4* from gDNA and cDNA are shown on the agarose gel. Lane 1: amplification of *ron4* from gDNA. Lane 2: amplification of *ron4* from a sample of RNA from VCG-1 strain schizonts treated with reverse transcriptase (RT+). Lane 3: amplification of *ron4* from a sample of RNA from VCG-1 schizonts without reverse transcriptase (RT-). MWM: molecular weight marker. Lane 4. Amplification of a *pvron1* fragment from an RT+ sample. Lane 5: *pvron1* amplification from an RT- sample.

Based on bioinformatics analysis, the PVX_091435 gene ID gene was re-annotated (Figure 1) and such information was used to design primers for amplifying *pvron4* from gDNA and cDNA. *pvron4*G1 5’- ATG TCT CGT AAA AGG GTT TT-3’ and *pvron4*G2 5’-CAA GTC TTC AAA AAT GAG ATT T-3’ primers amplified from predicted ATG until the stop codon. These primers were included in PCR reactions with Gotaq flexi DNA polymerase (Promega) with gDNA or RT-PCR products from samples treated with or without reverse transcriptase (RT^+^ and RT^-^ respectively) at 25 μL final volume, according to the manufacturer’s instructions. Amplification conditions were as follows: a 5-min cycle at 95°C, followed by 35 cycles of 1 min at 56°C, 3 min at 72°C and 1 min at 95°C. A final extension step lasted 10 min at 72°C. A *pvron1* region (~1053 from cDNA) transcribed during the erythrocyte phase was amplified using direct 5′- atg GCG AAG GAG CCC AAG TG-3′ and reverse 5′- ATC CCT AGC AAT GCT TCG -3′ primers for evaluating cDNA contamination by gDNA [36].

Primers called ExtD: 5’-TCC AGA CGT GTC AGA GTG-3’ and ExtR: 5’- TAG CTT CGT TCC TTT GGG-3’ were also designed; they were localized 72 bp upstream of the start codon and 49 bp downstream the stop codon of the *pvron4* gene, respectively. Amplification conditions from gDNA and cDNA were the same as those used for the *pvron4*G1 and *pvron4*G2 primers. The polymerase chain reaction (PCR) products were visualized on 1% agarose gel and then purified with a Wizard PCR preps kit (Promega). PCR products from gDNA and cDNA were cloned in pGEM-T (Promega) and pEXP5-CT/TOPO vectors (Invitrogen) respectively by TA cloning. Positive clones and PCR products were sequenced in an ABI PRISM 310 Genetic Analyzer. The sequences were analyzed using CLC DNA Workbench (CLC bio) and compared to the Sal-1, Mauritania I and India VII strain sequences [37] using Clustal W [38].

### Obtaining polyclonal antibodies against *Pv*RON4, *Pv*12 and *Pv*RON2

Taking the amino acid sequence predicted for *Pv*RON4 as reference, two 20 residue-long, B-epitope peptides were selected, chemically synthesized in solid phase and cysteine and glycine (CG) were added to amino and carboxy termini for polymerization. Synthetized peptides were numbered 36114 (CG^181^GSASESAAPSEEKASEENVP^200^GC) and 36115 (CG^719^AAEVEKRGFDEDYIQEEIKN ^738^GC). These peptides were selected based on high average values for Parker’s antigenicity, hydrophilicity and solvent accessibility using Antheprot software [39] and high values obtained when using Bepipred (0.35 default threshold and 75% specificity) [40]. It was also taken into account that the peptides corresponded to different protein regions, for detecting fragments in case of proteolytic processing occurring in *Pv*RON4.

Seven to eight week-old BAlb/C mice were intraperitoneally (ip) inoculated with 75 μg of each polymeric peptide emulsified in Freund’s complete adjuvant (FCA) on day 0 for evaluating *Pv*RON4 expression in VCG-1 strain schizonts. Three boosters were given on days 30, 45 and 60 using the same peptides emulsified in Freund’s incomplete adjuvant (FIA). Polyclonal antibodies against *Pv*12 localized on the membrane and *Pv*RON2 localized in the rhoptries were obtained for *Pv*RON4 localization studies. Peptide 35520 (CG^1674^KLQQEQNELNEEKERQRQEN^1693^GC) [8] was used for *Pv*RON2 and peptides CG-AKIRVRKRSGEEYDKEIFNL-GC and CG-AHFEFATTPDDQNSVSEPRA-GC were used for *Pv*12 [10]. These peptides were inoculated into negative New Zealand rabbits for recognizing *P. vivax-*derived proteins by Western blot, as described previously [8,10]. Animal sera was collected and stored at -70°C for later studies. Animal immunization and bleeding was done according to Colombian animal protection recommendations (Law 84/1989) and Resolution 8430/1993 for handling live animals for research or experimentation purposes.

### *Pv*RON4 immunodetection assays

#### ELISA

A previously described ELISA test was used for determining inoculated peptide immunogenicity [19]. Briefly, each of the inoculated peptides was sown in 96-well ELISA plates and incubated at 1:100 dilution of each serum. A peroxidase-coupled, anti-mouse antibody was used as secondary antibody (Vector Laboratories), diluted 1:5,000.

#### Western blot

The proteins were extracted from a *P. vivax* schizont-enriched sample and separated on an SDS-PAGE gel, as previously described [8]. Proteins transferred to a nitrocellulose membrane were incubated with pre-immune or immune serum. In a parallel assay, immune serum was pre-incubated with inoculated polymeric peptides before being incubated with the nitrocellulose membrane. A peroxidase-coupled, anti-mouse IgG antibody was used as secondary antibody (Vector Laboratories).

#### Indirect immunofluorescence

A suspension of erythrocytes parasitized by *P. vivax* VCG-1 was washed thrice with PBS (pH 7.2–7.4), then centrifuged at 2,500×g for 4 min and suspended in a 1:1 PBS–fetal bovine serum solution. This solution was seeded on eight-well multitest slides and left to dry at room temperature for 24 hr. Parasites slides were fixed using a 4% formaldehyde solution followed by three washes with PBS. The slides were blocked with 1% skimmed milk in PBS for 30 min and incubated with anti-*Pv*RON4 primary antibody obtained in mice and anti-*Pv*12 or anti-*Pv*RON2 antibodies in 1:40 dilution. Fluorescein-labelled anti-rabbit IgG (FITC) (Vector Laboratories) and rhodamine-labelled anti-mouse IgG (Millipore) were used as secondary antibody for 60 min, followed by three PBS washes. Parasite nuclei were stained with a 2 μg/mL solution of 4’,6-diamidino-2-phenylindole (DAPI) for 20 min at room temperature and fluorescence was visualized in a fluorescence microscope (Olympus BX51) using an Olympus DP2 camera and Volocity software (Perkin Elmer).

### Statistical analysis

Differences in antibody production between pre-immune and post-third immunization sera obtained from ELISA were evaluated using non-parametric Wilcoxon signed rank test.

## Results and discussion

### In-silico re-annotation of the *ron4* gene in *Plasmodium vivax*

The search for the *pvron4* gene was initially made using the *Pf*RON4 protein amino acid sequence (PF11_0168) containing 1,201 residues [18]. tBlastn results revealed a nucleotide region having a high probability of containing *pvron4* in the PviS_CM000450 contig localized in chromosome 9. Analysis showed a *pvron4* segment running from nucleotide 516,555 to 514,248 corresponding to residues 484 to 1,201 in *P. falciparum*; however, the beginning of *pvron4* could not be found. The *P. knowlesi* RON4 (PKH_091340) amino acid sequence reported in PlasmoDB was used to overcome this. *Plasmodium knowlesi* is a parasite which infects *Macaca fascicularis* monkeys and shares a close phylogenetic relationship with *P. vivax* as shown in small subunit rRNA analysis and the high identity and similarity values between orthologous proteins [9,41]. This led to finding that *pvron4* was located between nucleotide 516,904 and 514,248, having a ~2,657 bp length. Interestingly, a putative gene called PVX_091435 (8,437 bp and six exons) was found to be annotated in this nucleotide region which is also found when *Pf*RON4 and *Pk*RON4 amino acid sequences are used as templates for Blastp analysis (Figure 1). However, analyzing the presence of and boundaries for the exons/introns proposed for the PVX_091435 gene indicated that its gene structure had not been correctly annotated (Figure 1). In-silico analysis of this genome locus suggested that there were two ORFs; the first had high similarity (60%) with a *P. falciparum* serine esterase and began at position 522,681 of the PviS_CM000450 contig and ended at nucleotide 518,314 (Figure 1) while the second ORF was found downstream serine esterase in position 516,904 and referred to *pvron4*. The suggested *pvron4* structural region had three exons; a first short one ~78 bp (in ORF -1) having consensus intron splice sites, a second long ~2,019 bp (in ORF -2) having its respective donor site sequence ending with a ~189 bp exon (in ORF -3) (Figure 1).

**Figure 2:**
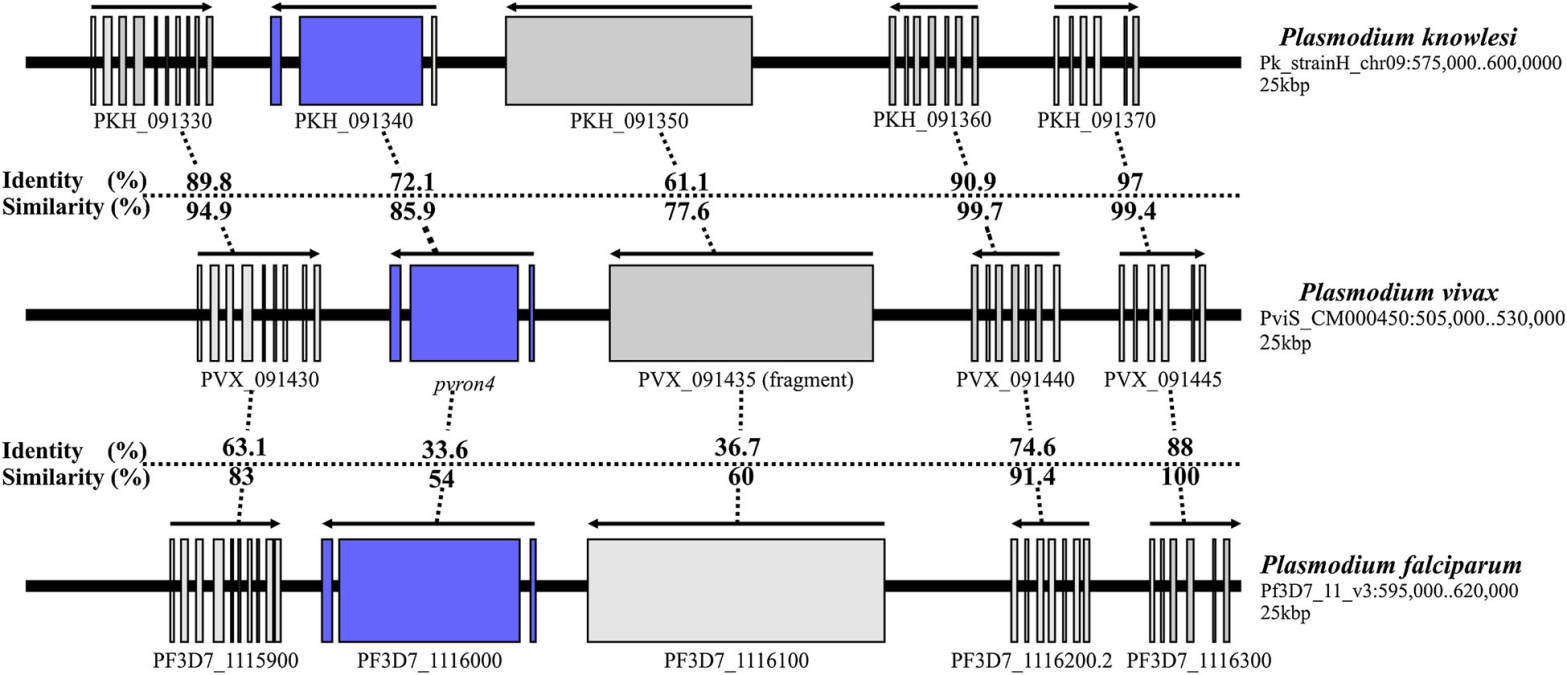
**Syntenic analysis of the chromosome region containing *ron4 *in *Plasmodium knowlesi, Plasmodium falciparum *and *Plasmodium vivax.*** Blue shows *ron4* location, grey shows open reading frames (ORFs) upstream and downstream *ron4*. Each ORF’s accession number in PlasmoDB is indicated, as well as amino acid similarity and identity levels between *Pf*-*Pv* and *Pv*-*Pk*.

Once *ron4* structure had been annotated, a syntenic analysis was made for evaluating neighboring genes’ structure (number of introns and exons), transcription orientation and determining identity (ID) and similarity (SI) values at protein level. *Pv*RON4 amino acid alignment with *Pf*RON4 and *Pk*RON4 showed that ID and SI values were greater with *Pk*RON4 (Figure 2) thereby agreeing with the close relationship between these two parasite species. This coincided with previous studies showing greater similarity between *P. vivax* and *P. knowlesi* proteins than with other *Plasmodium* species [10,11]. Conservation was also found regarding the structure of genes upstream and downstream *ron4* in *P. falciparum*, *P. vivax* and *P. knowlesi*, having 61.1%-97% and 77.6%-99.7% amino acid ID and SI values for *Pk-Pv* respectively, and 33.6%-88% and 54%-100% for *Pf-Pv*, respectively (Figure 2).

### Transcription and analysis of the VCG-1 strain *pvron4* sequence

The *pvron4* gene was amplified from gDNA and cDNA to confirm its presence and re-annotation. Figure 1 shows that a ~2,600 bp gDNA product was obtained and a ~2,200 bp product from cDNA (RT+) thereby confirming the presence of intron regions. No amplification was observed from RT- samples, indicating that the cDNA preparation had no gDNA contamination. PCR amplification of the *pvron4* encoding sequence suggested the presence of this transcript (~2,200 bp) in mature *P. vivax* blood stages, similar to that found for *pvron1* (Figure 1) [36]. The *pvron4* transcription time coincided with that displayed by *pfron4* (42-48 hr of the intra-erythrocyte cycle) [42]. Transcriptome and proteome analysis of *P. falciparum* has shown that most ORFs transcribed at the end of the intra-erythrocyte cycle are expressed during and participate in host cell invasion [42,43].

**Figure 3:**
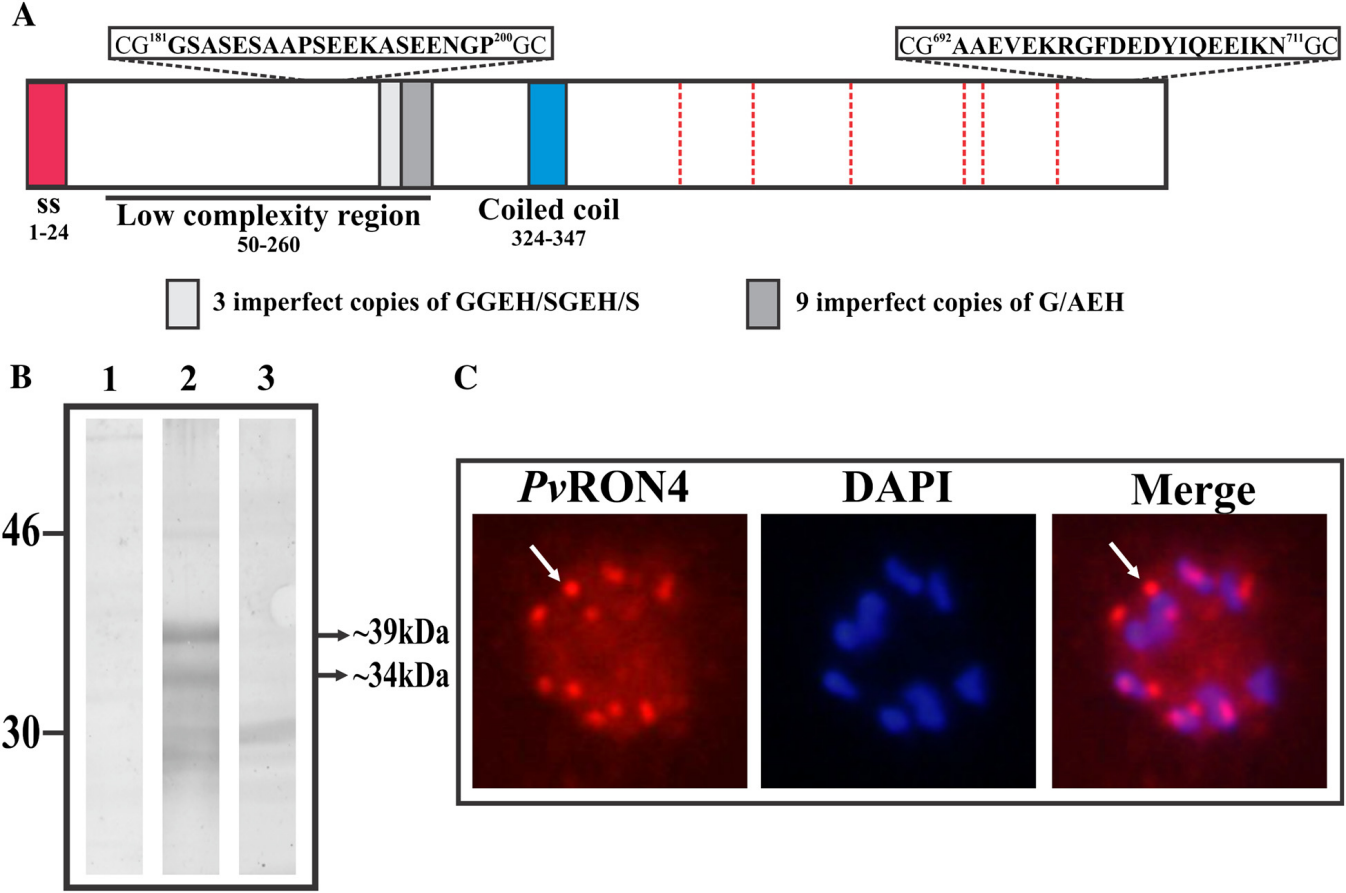
***Pv*RON4 expression in VCG-1 schizonts. (A)** Schematic representation of *Pv*RON4. The most relevant characteristics in the protein’s primary sequence are shown. Red shows signal peptide location, blue a coiled-coil α-helical motif and grey the tandem repeats. Red lines indicate conserved cysteines between Sal-1, VCG-1, Mauritania I and India VII strains. Inoculated peptides’ localization and sequence are shown in the boxes. **(B)***Pv*RON4 recognition in schizont lysate. Lane 1: pre-immune serum; lane 2: immune serum; lane 3: immune serum pre-incubated with inoculated peptides. **(C)** Immunofluorescence studies on schizonts. The arrows indicate the presence of the protein (red) and the apical pattern suggesting its localization in the apical organelles. Blue shows schizont nuclei stained with DAPI.

When aligning *pvron4* gDNA and cDNA nucleotide sequences obtained from sequencing three independent clones it was found that the gDNA was 2,573 bp while cDNA was 2,202 bp. The gene consisted of three exons and two intron regions, encoding a 733 residue-long protein (~80 kDa); this genomic organization coincided with that proposed by bioinformatics analysis (Figure 1). When comparing the sequences obtained from VCG-1 to those reported for Sal-1 (PVX_091435), a match was found from nucleotide 516,904 onwards within the contig, coinciding with previous analysis proposed for *pvron4* localization and structure (Figure 1). The alignment showed that exon 2 was shorter in VCG-1 (1,932 bp) compared to Sal-1 (2,019 bp) due to deletions corresponding to a loss of 81 nt (27 amino acid residues) between amino acids 235-262 and a deletion of two triplets (GTG AGG) encoding glycine (G671) and glutamic acid (E672). According to bioinformatics analysis, exon two encoded a low complexity region rich in tandem repeats (TR) (Figure 3). Analysis also revealed a change at nucleotide level involving T for G in position 810 (T810G); this substitution led to a valine (V) being changed for a glycine (G) in position 199 (V199G). A substitution of A for G was found in VCG-1 strain intron 2 compared to Sal-1. VCG-1 gDNA and cDNA consensus sequences were deposited in [GenBank: KF378614 and KF378615].

Comparative analysis between VCG-1 *Pv*RON4 (Colombian), Sal-1 (El Salvador), India VII (southern Asia) and Mauritania I (Africa) amino acid sequences revealed differences regarding protein length (see Additional file 1), mainly due to large deletions between residues 179-305 where the TRs occurred. The first 150 amino acids corresponded to a conserved region followed by a variable zone having repeat regions and a highly conserved carboxy-terminal in the strains evaluated here. Such distribution was similar to that reported for several antigens proposed as candidates for an anti-*P. falciparum* vaccine where proteins’ conserved regions are surrounded by variable and low complexity regions acting as immune system distractors, while important functional regions for the parasite remain poorly recognized. The circumsporozoite protein (CSP) consists of a central repeat region, which is diverse across *Plasmodium* species, and flanking the repeats are two conserved domains: region I, at the N-terminus of the repeats, and a known C-terminal cell-adhesive adhesive motif, termed type I thrombospondin repeat (TSR) [44]. Even though the repeat regions’ function has not been made clear to date, it has been found that they are species-specific, low complexity and (in many cases) are responsible for the diversity between different parasite isolates [45]. It has been suggested that the presence of polymorphic repeats affects antibody affinity maturation, thus suppressing antibody response to critical epitopes or adjacent regions [46].

### Bioinformatics analysis of *Pv*RON4 primary sequence

The *Pv*RON4 amino acid sequence was scanned for predicting different characteristics. VCG-1 *Pv*RON4 had a signal peptide within the first 24 amino acids (Figure 3A) and a probable type I peptidase cleavage site between residues S24-F25, suggesting that once the protein has been synthesized, it is transported to be either secreted or inserted into cell membranes. In fact, it has been reported that *Pf*RON4 and *Tg*RON4 homologous proteins are secreted by the parasite towards host cell membrane [47,48]. Bioinformatics analysis detected no potential transmembrane domains or GPI anchors in *Pv*RON4, *Tg*RON4 and *Pf*RON4, thereby classifying them as soluble proteins. It has been shown that some soluble *P. falciparum* merozoite surface proteins, such as MSP 3, 6, 7 and 9, are associated and/or interact with membrane-anchored antigens [49]. Interestingly, *Pv*RON4 contained a coiled-coil α-helical motif in its sequence between residues 324-347 (Figure 3A) suggesting *Pv*RON4 participation in protein-protein interaction and complex formation. A low complexity (Figure 3A), highly variable region was found (see Additional file 1) between residues 50 to 260 containing two TRs. The first repeat consisted of seven amino acids (GGEH/SGEH/S) and the second of three amino acids having nine imperfect copies of G/AEH residues. A conserved region between residues 277 and 733 was found when comparing RON4 from VCG-1 with other *P. vivax* strains, such region being characterized by having six conserved cysteines. Several functionally relevant malarial antigens, such as AMA-1 and RON2, have cysteine-containing regions forming disulphide bridges and/or ordered 3D structures for fulfilling their biological function [21]. However, further structural and functional studies are needed for evaluating this conserved domain’s importance in *Pv*RON4 and the influence of repeat regions in an immune response against *Pv*RON4.

### *Pv*RON4 expression and localization in VCG-1 late-stage schizonts

Anti-*Pv*RON4 antibodies were induced in mice for evaluating *Pv*RON4 expression and cell localization in *P. vivax* schizonts. ELISA revealed that immune sera showed high reactivity against 35114 and 35115 peptides (A_620_ 0.63 ± 0.22) compared to pre-immune sera (A_620_ 0.03 ± 0.01) (p=0.00). Sera ability to recognize *Pv*RON4 in VCG-1 schizont lysate was then evaluated using Western blot. Figure 3B shows that polyclonal antibodies specifically recognized ~39 kDa and ~34 kDa bands which were not recognized by either the pre-immune sera or hyper-immune sera which had been pre-incubated with the inoculated peptides. Taking a ~80 kDa predicted *Pv*RON4 size, two bands below the expected molecular weight were detected, suggesting that *Pv*RON4 undergoes a similar proteolytic processing to that reported for most proteins localized in the apical organelles, including other RON proteins [47]. It has been suggested that rhoptry proteins’ proteolytic cleavage is an essential event for maturation and/or promoting interaction with cell surface receptors or between parasite proteins [47,50].

**Figure 4:**
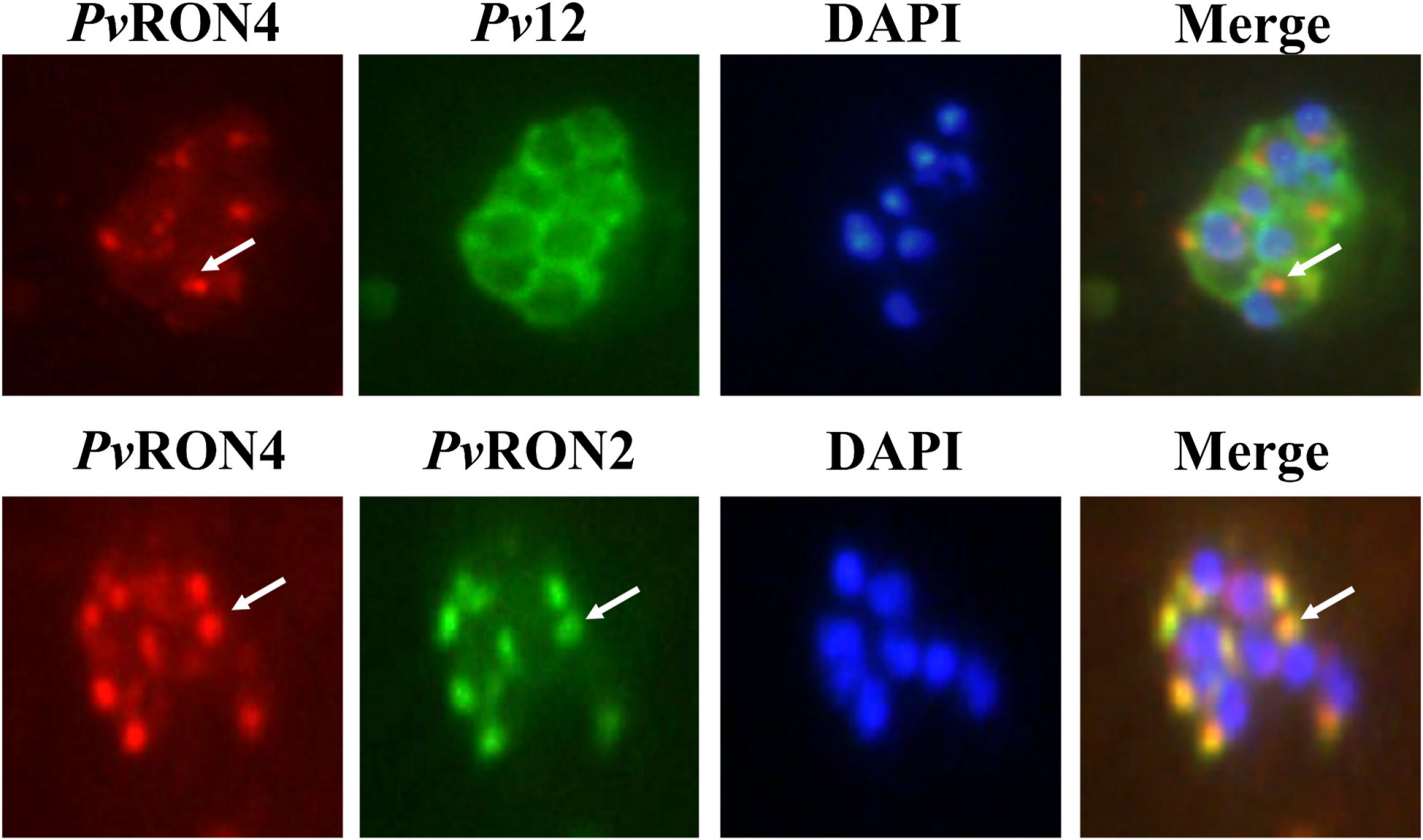
***Pv*RON4 co-localization studies with *Pv*12 and *Pv*RON2.** Anti-*Pv1*2 and anti-*Pv*RON2 antibodies were used for determining the approximate localization of *Pv*RON4 in late stage VCG-1 schizonts. As seen in the photo, the *Pv*RON4 signal did not overlap with the *Pv*12 membrane protein fluorescence pattern, while showing overlapping with the pattern for the rhoptries obtained with the anti-*Pv*RON2 antibody. The arrows indicate *Pv*RON4 presence.

The anti-*Pv*RON4 antisera also recognized the protein on VCG-1 late-stage schizonts, having the typical apical organelle punctate pattern (Figure 3C) common to other apical proteins [8,18]. Co-localization studies using antibodies against *Pv*RON4 and *Pv*12 showed that while *Pv*12 was expressed on merozoite surface with a bunch of grapes-like pattern, *Pv*RON4 had a punctate pattern, not overlapping *Pv*12 staining (Figure 4). When anti-*Pv*RON4 antibodies were used with anti-*Pv*RON2, the staining overlapped, coinciding with *Tg*RON4 and *Pf*RON4 localization in the rhoptry neck (Figure 4). Previous studies in *T. gondii* and *P. falciparum* have shown that RON4 is the only protein where strong evidence has been produced concerning localization in the TJ [25,51] and has been chosen as a model for studying the dynamics of merozoite invasion of RBC [25].

## Conclusions

Bioinformatics and experimental analyses allowed the identification of a new gene having three exons and encoding the *Pv*RON4 protein. Like *Pf*RON4, its *P. falciparum* homologue, *Pv*RON4 is expressed in late schizonts and is localized in the rhoptries. Strong evidence regarding RON4 association in the TJ and its conservation in the *Plasmodium* genus has highlighted this protein’s importance and the need for evaluating its mechanism of action and association within the *P. vivax* AMA1-RON complex.

## Competing interests

The authors declare that they have no competing interests.

## Authors’ contributions

GAP carried out bioinformatics analyses, molecular biology assays and wrote the initial manuscript. HC synthesized and purified the peptides used for rabbit and mice immunizations and analyzed data. JA carried out immunoassays. MAP evaluated and coordinated assays, and revised the final manuscript. All authors read and approved the final manuscript.

## Supplementary Material

Additional file 1 ***Pv*RON4 amino acid alignment between different *Plasmodium vivax* strains.** Conserved cysteines between VCG-1 (Colombia), Sal-1 (Salvador), India VII (India) and Mauritania I (Africa) strains are shown in red. Click here for file
