# Supplementary material for: Annotation and characterization of the Plasmodium vivax rhoptry neck protein 4 (PvRON4)
Source: Malar J. 2013 Oct 5;12:356. doi: 10.1186/1475-2875-12-356 (PMC3851475; doi:10.1186/1475-2875-12-356)
Supplement: Additional file 1 — PvRON4 amino acid alignment between different Plasmodium vivax strains. Conserved cysteines between VCG-1 (Colombia), Sal-1 (Salvador), India VII (India) and Mauritania I (Africa) strains are shown in red. [file 1475-2875-12-356-S1.rtf]

                                                                                                                             
                      *        20         *        40         *        60         *        80         *       100            
Sal-1      : MSRKRVFLLCIFLAISAVVDEAESFRKPDAAANVSFLEFTADGNAEEGQAQPSEPIVNTQPLQENVSEAAPHSEESTGGSAEKGSGGHEETPHAGEHDKGSGGSNG : 106
VCG-1      : MSRKRVFLLCIFLAISAVVDEAESFRKPDAAANVSFLEFTADGNAEEGQAQPSEPIVNTQPLQENVSEAAPHSEESTGGSAEKGSGGHEETPHAGEHDKGSGGSNG : 106
India VII  : MSRKRVFLLCIFLAISAVVDEAESFRKPDAAANVSFLEFTADGNAEEGQAQPSEPIVNTQPLQENVSEAAPHSEESTGGSAEKGSGGHEETPHAGERDKGSGGSNG : 106
Mauritania : MSRKRVFLLCIFLAISAVVDEAESFRKPDAAANVSFLEFTADGNAEEGQAQPSEPIVNTQPLQENVSEAAPHSEESTGGSAEKGSGGHEETPHAGEHDKGSGG--- : 103

                                                                                                                             
                *       120         *       140         *       160         *       180         *       200         *        
Sal-1      : SSESNGSVLADGHAEEAPNEQVSSEKNDGHSESSTTGSHSDKEELDLYTSSPSDKEENSAPAMGGMHNTVG-----------LRGGSASESAAPSEEKASEENVPH : 201
VCG-1      : SSESNGSVLADGHAEEAPNEQVSSEKNDGHSESSTTGSHSDKEELDLYTSSPSDKEENSAPAMGGMHNTVG-----------LRGGSASESAAPSEEKASEENGPH : 201
India VII  : SSESNGSVLADGHAEEAPNEQVSSEKNDGHSESSTTGSHSDKEELDLYTSSPSDKEENSAPAMGGMHNTRKHPKRMSPMKWQIKMGRTPHRRVPTRERCHLRMWVA : 212
Mauritania : ---SNGSVLADGHAEEAPNEQVSSEKNDGHSESSTTGSHSDKEELDLYTSSPSDKEENSAPAMGGMHNTRKHPKRMSPMKWQIKMGRTPHRRVPTRERCHLRMWVA : 206

                                                                                                                             
                  220         *       240         *       260         *       280         *       300         *       3      
Sal-1      : EVAD--KNGEDTSQEGSHAGALSPAHVGGESGESGGEHGEHGEHAEHGEHGEHGEHGEHAEHGEHGEHGEHAEHGEHGEHGEHGEHGEHAEQEEHQIYGTIHPEAE : 305
VCG-1      : EVAD--KNGEDTSQEGSHAGALSPAHVGGESGESGGEHGEHGEHAEHGEHG---------------------------EHGEHGEHGEHAEQEEHQIYGTIHPEAE : 278
India VII  : RVAASTESTVNTLNMGSTESTVN-----------------------------------------------------------------------MASTVNMASTAE : 247
Mauritania : RVAASTESTVNTLNMGSTESTVNTLNMGSTESTVNMV--------------------------SMVSTVSMVSMVSTVNMVSMGSTVSTLNMGSTESTVNMASTAE : 286

                                                                                                                             
             20         *       340         *       360         *       380         *       400         *       420          
Sal-1      : ASILPALKEKGIHVSSPERVILEIIESAKEGIKGLLKLRKSKDTGKLLEEALEKLNINQRDLHANNNFITLEMYDRILSEMFKILTEMSFYKDGHFYETLGLNKSI : 411
VCG-1      : ASILPALKEKGIHVSSPERVILEIIESAKEGIKGLLKLRKSKDTGKLLEEALEKLNINQRDLHANNNFITLEMYDRILSEMFKILTEMSFYKDGHFYETLGLNKSI : 384
India VII  : ASILPALKEKGIHVSSPERVILEIIESAKEGIKGLLKLRKSKDTGKLLEEALEKLNINQRDLHANNNFITLEMYDRILSEMFKILTEMSFYKDGHFYETLGLNKSI : 353
Mauritania : ASILPALKEKGIHVSSPERVILEIIESAKEGIKGLLKLRKSKDTGKLLEEALEKLNINQRDLHANNNFITLEMYDRILSEMFKILTEMSFYKDGHFYETLGLNKSI : 392

                                                                                                                             
                  *       440         *       460         *       480         *       500         *       520         *      
Sal-1      : LNQSLKEIKIKMLRTIGVPYTKLPPIVKNKEKESTCAANNLIISITSKELAQRMAIMFAKWLAPEEYGSVVDLDKSIELNVLCAGAPILVQQWKYYQNMLGFETGN : 517
VCG-1      : LNQSLKEIKIKMLRTIGVPYTKLPPIVKNKEKESTCAANNLIISITSKELAQRMAIMFAKWLAPEEYGSVVDLDKSIELNVLCAGAPILVQQWKYYQNMLGFETGN : 490
India VII  : LNQSLKEIKIKMLRTIGVPYTKLPPIVKNKEKESTCAANNLIISITSKELAQRMAIMFAKWLAPEEYGSVVDLDKSIELNVLCAGAPILVQQWKYYQNMLGFETGN : 459
Mauritania : LNQSLKEIKIKMLRTIGVPYTKLPPIVKNKEKESTCAANNLIISITSKELAQRMAIMFAKWLAPEEYGSVVDLDKSIELNVLCAGAPILVQQWKYYQNMLGFETGN : 498

                                                                                                                             
                    540         *       560         *       580         *       600         *       620         *            
Sal-1      : EHAFLNLIDELLVIDKRHSNNEAYSKVIRKIKKSKAFNYCTKVMRIAGNISSIPFNHENNKTPSYSIIGSLGNLVKAHMGNYYVAIANRINSYFAYAEKRNKKNSP : 623
VCG-1      : EHAFLNLIDELLVIDKRHSNNEAYSKVIRKIKKSKAFNYCTKVMRIAGNISSIPFNHENNKTPSYSIIGSLGNLVKAHMGNYYVAIANRINSYFAYAEKRNKKNSP : 596
India VII  : EHAFLNLIDELLVIDKRHSNNEAYSKVIRKIKKSKAFNYCTKVMRIAGNISSIPFNHENNKTPSYSIIGSLGNLVKAHMGNYYVAIANRINSYFAYAEKRNKKNSP : 565
Mauritania : EHAFLNLIDELLVIDKRHSNNEAYSKVIRKIKKSKAFNYCTKVMRIAGNISSIPFNHENNKTPSYSIIGSLGNLVKAHMGNYYVAIANRINSYFAYAEKRNKKNSP : 604

              

                                                                                                               
              640         *       660         *       680         *       700         *       720         *       740        
Sal-1      : LKVVSVCTLLHLTDMLHNCSDEHLKNILDLNTLKLNILNMQGRRVLQPLVKMSFLGAAQNPALKEICEPSNHLVGEDETLSKLLNLLSTGSHELLAAEVEKRGFDE : 729
VCG-1      : LKVVSVCTLLHLTDMLHNCSDEHLKNILDLNTLKLNILNMQGRRVLQPLVKMSFLGAAQNPALKEICEPSNHLV--DETLSKLLNLLSTGSHELLAAEVEKRGFDE : 700
India VII  : LKVVSVCTLLHLTDMLHNCSDEHLKNILDLNTLKLNILNMQGRRVLQPLVKMSFLGAAQNPALKEICEPSNHLV--DETLSKLLNLLSTGSHELLAAEVEKRGFDE : 669
Mauritania : LKVVSVCTLLHLTDMLHNCSDEHLKNILDLNTLKLNILNMQGRRVLQPLVKMSFLGAAQNPALKEICEPSNHLV--DETLSKLLNLLSTGSHELLAAEVEKRGFDE : 708

                                                     
                    *       760         *            
Sal-1      : DYIQEEIKNINESDNNVRDKGEDEVENLIFEDL- : 762
VCG-1      : DYIQEEIKNINESDNNVRDKGEDEVENLIFEDL- : 733
India VII  : DYIQEEIKNINESDNNVRDKGEDEVENLIFEDL- : 702
Mauritania : DYIQEEIKNINESDNNVRDKGEDEVENLIFEDL- : 741
